# The Suprachiasmatic Nucleus of the Dromedary Camel (*Camelus dromedarius*): Cytoarchitecture and Neurochemical Anatomy

**DOI:** 10.3389/fnana.2017.00103

**Published:** 2017-11-16

**Authors:** Khalid El Allali, Mohamed R. Achaâban, Mohammed Piro, Mohammed Ouassat, Etienne Challet, Mohammed Errami, Nouria Lakhdar-Ghazal, André Calas, Paul Pévet

**Affiliations:** ^1^Comparative Anatomy Unit/URAC49, Department of Biological and Pharmaceutical Veterinary Sciences, Hassan II Agronomy and Veterinary Medicine Institute, Rabat, Morocco; ^2^PMC-EC, Department of Medicine, Surgery and Reproduction, Hassan II Agronomy and Veterinary Medicine Institute, Rabat, Morocco; ^3^Neurobiology of Rhythms UPR 3212 CNRS, Institute for Cellular and Integrative Neurosciences, University of Strasbourg, Strasbourg, France; ^4^Department of Biology, Faculty of Science, Abdelmalek Essaâdi University, Tétouan, Morocco; ^5^Unit of Research on Biological Rhythms, Neuroscience and Environment, Faculty of Science, Mohammed V-Agdal University, Rabat, Morocco; ^6^IINS, CNRS UMR 5297, University of Bordeaux, Bordeaux, France

**Keywords:** suprachiasmatic nucleus, immunofluorescence, cytoarchitecture, neuropeptides, oxytocin, tyrosine hydroxylase, dromedary camel

## Abstract

In mammals, biological rhythms are driven by a master circadian clock located in the suprachiasmatic nucleus (SCN) of the hypothalamus. Recently, we have demonstrated that in the camel, the daily cycle of environmental temperature is able to entrain the master clock. This raises several questions about the structure and function of the SCN in this species. The current work is the first neuroanatomical investigation of the camel SCN. We carried out a cartography and cytoarchitectural study of the nucleus and then studied its cell types and chemical neuroanatomy. Relevant neuropeptides involved in the circadian system were investigated, including arginine-vasopressin (AVP), vasoactive intestinal polypeptide (VIP), met-enkephalin (Met-Enk), neuropeptide Y (NPY), as well as oxytocin (OT). The neurotransmitter serotonin (5-HT) and the enzymes tyrosine hydroxylase (TH) and aromatic L-amino acid decarboxylase (AADC) were also studied. The camel SCN is a large and elongated nucleus, extending rostrocaudally for 9.55 ± 0.10 mm. Based on histological and immunofluorescence findings, we subdivided the camel SCN into rostral/preoptic (rSCN), middle/main body (mSCN) and caudal/retrochiasmatic (cSCN) divisions. Among mammals, the rSCN is unusual and appears as an assembly of neurons that protrudes from the main mass of the hypothalamus. The mSCN exhibits the triangular shape described in rodents, while the cSCN is located in the retrochiasmatic area. As expected, VIP-immunoreactive (ir) neurons were observed in the ventral part of mSCN. AVP-ir neurons were located in the rSCN and mSCN. Results also showed the presence of OT-ir and TH-ir neurons which seem to be a peculiarity of the camel SCN. OT-ir neurons were either scattered or gathered in one isolated cluster, while TH-ir neurons constituted two defined populations, dorsal parvicellular and ventral magnocellular neurons, respectively. TH colocalized with VIP in some rSCN neurons. Moreover, a high density of Met-Enk-ir, 5-HT-ir and NPY-ir fibers were observed within the SCN. Both the cytoarchitecture and the distribution of neuropeptides are unusual in the camel SCN as compared to other mammals. The presence of OT and TH in the camel SCN suggests their role in the modulation of circadian rhythms and the adaptation to photic and non-photic cues under desert conditions.

## Introduction

Rhythmicity is a ubiquitous property of all living organisms (Turek and Van Reeth, 1996). Biological rhythms in mammals are driven by a circadian clock located in the suprachiasmatic nucleus (SCN) of the hypothalamus (Moore and Eichler, 1972; Stephan and Zucker, 1972; Inouye and Kawamura, 1979; Green and Gillette, 1982; Groos and Hendriks, 1982; Lehman et al., 1987). The SCN is a strong autonomous oscillator cycling with a period close to, but different from, 24 h and entrained to exactly 24 h by environmental cues, the *Zeitgeber* (Refinetti, 2006). This nucleus is a complex structure containing several neuronal populations that have specific afferents and efferents. In most species, especially in rodents, the SCN is divided into two subdivisions: the dorsomedial SCN or ‘shell’ and the ventrolateral SCN or ‘core’ (Abrahamson and Moore, 2001; Morin et al., 2006). The dorsomedial SCN contains neuronal perikarya immunopositive for arginine-vasopressin (AVP) (Ibata et al., 1999; Moore et al., 2002; Morin et al., 2006; Nascimento et al., 2010) while the ventrolateral SCN contains neurons expressing vasoactive intestinal polypeptide (VIP), gastrin releasing peptide and peptide histidine isoleucine (Stopa et al., 1984; Card et al., 1988; Mikkelsen et al., 1991; Swaab et al., 1994; Smale and Boverhof, 1999; Moore et al., 2002; Nascimento et al., 2010). A large number of other neuropeptides have been described in the SCN of mammals with some interspecies variations. It was demonstrated that somatostatin perikarya are generally located in the intermediate region of SCN (Card et al., 1988; Ibata et al., 1999) while neurophysin, neurotensin, thyrotropin-releasing hormone, enkephalins (Enk), and angiotensin II were described in different parts of the nucleus (Block et al., 1988; Tillet et al., 1989; Abrahamson and Moore, 2001; Thomas et al., 2004). Moreover, calcitonin gene-related peptide (Park et al., 1993), galanin (Skofitsch and Jacobowitz, 1985; Abrahamson and Moore, 2001), substance P (Morin et al., 1992; Mikkelsen and Larsen, 1993; Abrahamson and Moore, 2001; Piggins et al., 2001) and the calcium-binding protein calbindin (Silver et al., 1996; Ikeda and Allen, 2003; Menet et al., 2003) have also been reported in this nucleus in different species. Additionally, gamma-aminobutyric acid (GABA), an important neurotransmitter in the circadian system, is found in almost all neurons of the SCN (Okamura et al., 1989; Moore and Speh, 1993; Kalsbeek et al., 2000; Moore et al., 2002).

In addition to neuronal cell bodies, a typically dense innervation for various neuroactive agents exists in the SCN. Glutamate and pituitary adenylate cyclase activating peptide afferents originating from melanopsin retinal ganglion cells and forming the retinohypothalamic tract (RHT) are observed in the ventral region of the SCN containing VIP neurons (for review see Hannibal, 2002). Moreover, the ventrolateral SCN of rodents also contains serotonin (5-HT)-immunoreactive (ir) fibers that originate from the midbrain raphe nuclei. The core of the SCN also receives afferents originating from the intergeniculate leaflet (IGL) of the lateral geniculate complex containing neuropeptide Y (NPY), GABA and met-enkephalin (Met-Enk) (Moore and Eichler, 1972; Moore et al., 1978; Steinbush, 1981; van den Pol and Tsujimoto, 1985; Card and Moore, 1988, 1989; Tillet et al., 1989; Morin et al., 1992).

Functional relevance of the neurochemical composition of the master clock has not yet been understood fully. However, mechanisms and molecular processes underlying the synchronization of the SCN by environmental cues are well documented. It is known that in mammals, the light-dark cycle (LD) is the strongest *Zeitgeber* (Refinetti, 2006) imposing its period and phase to the circadian clock. The VIP neurons of the ventrolateral SCN, which are the target of the RHT (Tanaka et al., 1993), play an important role in the synchronization by light and transmit photic signaling to the pacemaker AVP neurons located dorsally in the dorsomedial SCN (Jacomy et al., 1999; Aton et al., 2005). In mammals, the molecular basis of clock function includes alternating activation and repression of gene expression by different proteins. These rhythmic self-regulating proteins are encoded by clock genes. The molecular machinery of the main clock is well known, and is based on two interdependent feedback loops (Reppert and Weaver, 2000, 2001; Shearman et al., 2000; Okamura et al., 2002; Takahashi et al., 2008; Mohawk et al., 2012).

In addition to photic entrainment by the LD cycle, a non-photic entrainment by other *Zeitgebers* and cues also exists. In particular, these include ambient temperature (Ta), food availability, pharmacological effect of melatonin and social cues. Experimental lesions have demonstrated that the geniculohypothalamic tract (Janik and Mrosovsky, 1994; Wickland and Turek, 1994; Challet et al., 1996; Miller et al., 1996; Schuhler et al., 1999; Goel et al., 2000) and serotoninergic afferents from the raphe nucleus (Cutrera et al., 1994; Miller et al., 1996; Challet et al., 1997) are involved in such non-photic entrainment. The relative importance of non-photic *vs* photic entrainment has not been widely studied.

Recently, we have demonstrated in the dromedary camel (one-humped camel, *Camelus dromedarius*) that a non-photic entrainment can be sufficiently strong to drive the circadian clock (El Allali et al., 2013). Indeed, in this species, environmental temperature cycles as well as the LD cycle synchronize (phase and period) melatonin and body temperature rhythms. The neural circuitry and neuropeptides involved in such entrainment are still unknown. This raises several questions about the structure and functioning of the SCN in this species. However, there are no data on the organization of the camel hypothalamus and only limited data are available on the brain of this species. For these reasons, the present study aimed at investigating the organization of the SCN in the dromedary camel and intended to fill the existing gap in our understanding of neuroanatomy. We carried out a cartography of the nucleus and studied its cytoarchitecture. The distribution of relevant neuropeptides involved in the circadian system was also investigated. These include AVP, VIP, Met-Enk, NPY, and oxytocin (OT). The enzymes tyrosine hydroxylase (TH) and aromatic L-amino acid decarboxylase (AADC) and the neurotransmitter serotonin (5-HT) were also studied.

## Materials and Methods

This study was conducted by sampling the hypothalamus of 31 camels, slaughtered to provide meat for public consumption, at two sites: the Eddakhla slaughterhouse in the south of Morocco (latitude: 23^∘∘^43′ N, 15°57′ W) and the Rabat slaughterhouse in the north of the country (latitude: 34°01′ N, 6°50′ W). The animals included 13 females and 18 males of local varieties (Piro et al., 2011) aged between 4 and 12 years (the lifespan of a camel is 20–30 years). They were kept outdoors under natural environmental (photoperiod and temperature) conditions with free access to water. Slaughter was carried out at different periods of the year and almost at the same time of the morning: 06h00. Brain samples were dissected out as animals were slaughtered. The study was performed in conformity with the Hassan II Agronomy and Veterinary Institute of Rabat and Moroccan Ministry of Agriculture recommendations, which are in accordance with international ethical standards (Touitou et al., 2006).

The heads were perfused immediately after slaughter. The perfusion was performed through the two external carotids using, first, a washing heparin (2.5 UI/ml) solution in saline (0.9% sodium chloride, Fluka^®^), followed by tissue fixation (5–6 l), using either formaldehyde (Sigma–Aldrich^®^; 10% in water) for cytoarchitectonic studies or 4% paraformaldehyde (Scharlau^®^) in phosphate-buffered saline (PBS; 0.2M, pH 7.4) for immunohistochemistry. Thirty to forty minutes after perfusion, the brain was carefully removed and the hypothalamus dissected and post-fixed at 4°C for 3 days in the fixative solution.

### Histology

Fifteen hypothalami from both sexes were used for studying the SCN cytoarchitecture using Nissl and hematoxylin-eosin (H&E) stains of sections from paraffin-embedded tissue samples and frozen sections.

#### Paraffin Embedding

As mentioned above the brains were fixed using 10% formaldehyde solution. The hypothalamic samples containing the SCN were dehydrated, embedded in paraffin and cut into frontal or sagittal sections at a 6 or 9 μm thickness using a microtome (Shandon Hypercut^®^). All sections were collected and every 10th consecutive section was processed for staining. Freely floating sections in 4% warmed (42°C) gelatin solution were mounted on glass slides and allowed to dry for 20 min in an oven at 56°C. Slides were then immersed in toluene (two times for 5 min) and then stained either with H&E or Nissl staining (cresyl violet or toluidine blue). The staining procedure involved sequentially dipping the slides in different solutions: toluene and ethanol; and then dye solutions: hematoxylin (5 min) and eosin (5 min), or cresyl violet for 1 min or toluidine blue for 2 min. The staining was followed by washing in distilled water for 1 min, dehydration in different baths of ascending ethanol (75, 90, and 100%) and clearing with toluene.

#### Frozen Sections

These sections were used to calculate the length of the SCN and its subdivisions. The fixed hypothalamic specimens containing the SCN were cryoprotected in 20% sucrose (Fluka^®^) in distilled water until subsequent freezing in -30°C isopentane (C5H12, Fluka^®^) cooled with liquid nitrogen. Sections were cut in the coronal plane at a 20 μm thickness using a freezing microtome (Leica-3050^®^), mounted on gelatinized slides, and kept at -20°C until being processed for Nissl or H&E staining.

In all cases, sections were coated with mounting medium: Eukitt^®^ (Sigma–Aldrich) for cresyl violet and toluidine blue staining, and Canada balsam (Fisher Scientific) for H&E, and coverslipped. The sections were viewed under a light microscope (Topview 4500^®^) and images taken with Lumenera’s Lw1130-1.4 megapixel CCD digital camera.

### Immunofluorescence

Sixteen hypothalami of both sexes, perfused with 4% paraformaldehyde as indicated above, were used for the study of SCN chemical neuroanatomy. After post-fixation, hypothalamic tissue samples containing the SCN were rinsed three times for 10 min in phosphate buffer (PB 0.1M/ pH7.4), and then cryoprotected in 20% sucrose (Fluka^®^) in PB at 4°C until subsequent freezing in -30°C isopentane (C5H12, Fluka^®^) cooled with liquid nitrogen. The tissue samples were cut into 20 μm-thick sections using a freezing microtome (Leica-3050^®^). The sections were mounted on gelatin or Super-frost^®^ (Menzel Glaser) slides and kept at -20°C until being processed for immunofluorescence. For this procedure, the sections were pre-incubated for 1 h at room temperature in 5% bovine serum albumin (BSA, Sigma-Aldrich) and 0.5% of Triton (Sigma-Aldrich^®^) in 0.05M PBS. Incubation in the primary antibody was then carried out overnight at room temperature. The antibodies used are listed in **Table 1**.

**Table 1:** Primary antibodies used in the current study.

Antibodies	Raised in	Dilution	Origin
Polyclonal anti-AVP	Rabbit	1:1000	Gift of Dr. G. Alonso (Alonso, 1988).
Cyclic monoclonal anti-AVP	Mouse	1:500	Sigma^®^
Polyclonal anti-OT	Rabbit	1:500	Gift of Dr. G. Alonso (Alonso, 1988)
Polyclonal anti-VIP	Rabbit	1:1000	Gift of Dr. G. Tramu (Deloof et al., 1988
Monoclonal anti-TH antibody	Mouse	1:1000	Sigma^®^
Polyclonal anti-Met-enk	Rabbit	1:500	Gift of Dr. Michel Arluison (Tramu and Leonardelli, 1979
Polyclonal anti-5-HT	Rabbit	1:2000	Sigma^®^
Polyclonal anti-NPY	Rabbit	1:500	Gift of Dr. Michel Arluison (Ciofi et al., 1987)
Polyclonal anti-AADC	Rabbit	1:500	Biomol^®^

After being washed three times for 10 min in buffer-2 solution (0.2% BSA in 0.05M PBS), sections were incubated in the secondary antibody diluted in the same buffer-2 for 2 h at room temperature.

For single labeling, the secondary antibodies were biotinylated anti-rabbit IgGs raised in goat (Vector; diluted 1:200). After washes in 0.05M PBS, the sections were incubated in streptavidin-Cy3 (or streptavidin - FITC) diluted 1:200 in 0.05M PBS for 2 h in darkness. Sections were finally rinsed 4 times for 10 min in 0.05M PBS.

For double labeling, the secondary antibodies used were coupled directly to fluorochrome: anti-rabbit-Cy3 antibody raised in sheep (Sigma; diluted 1:500), anti-mouse-FITC antibody raised in horse (Vector, diluted 1:500) or anti-rabbit IgG Alexa Fluor 488 antibody raised in goat (diluted 1:200; Molecular Probes^®^). The sections were coverslipped in polyvinyl alcohol Moviwol^®^ 4-88 (Sigma–Aldrich). They were then examined using a fluorescence microscope (Leica^®^ DM400B), inverted fluorescent microscope with motorized stage (Zeiss^®^ Axiovert 200) or confocal microscope (Leica^®^ TCS SP2 AOBS).

Specificity tests were based on the omission of the primary or secondary antibodies in some sections. In all cases, the immunolabelling was completely abolished.

### Cytoarchitectural Study

The length of the SCN and its subdivisions were calculated as the sum of the cross-sectional surface area of each nucleus multiplied by the section thickness (20 μm) (Tessonneaud et al., 1994). Values were measured from frozen sections. Due to the large dimension of the camel hypothalamus, the frozen sectioning allowed a successful production of serial sections. Values from all specimens were averaged and expressed as a mean ± standard error mean (SEM).

The cell size, which corresponds to the widest diameter of the soma, was measured microscopically using two validated methods (Cassone et al., 1988; Kraus and Wolff, 2008; Mesaros et al., 2008; Suzuki et al., 2010): automatically by using Axovision^®^ software of Zeiss microscopy, or manually by using a metric ruler. The cell size (d) was calculated using the formula d = f/N where (f) is the field of view and (N) the estimated number of cells which fit across the diameter of the field of view. The calculation was limited to neurons with a visible nucleus in the focal plane. A total of 30 neurons were randomly selected from each specimen and data for each part of the SCN were averaged and expressed as mean ± SEM.

### Images

Due to the large dimensions of the camel hypothalamus, data are illustrated by images at different magnifications or collating images to reconstruct the SCN and surrounding structures. The montage was achieved automatically when a motorized microscope stage was available.

## Results

### SCN Morphology and Cytoarchitecture

**FIGURE 1:**
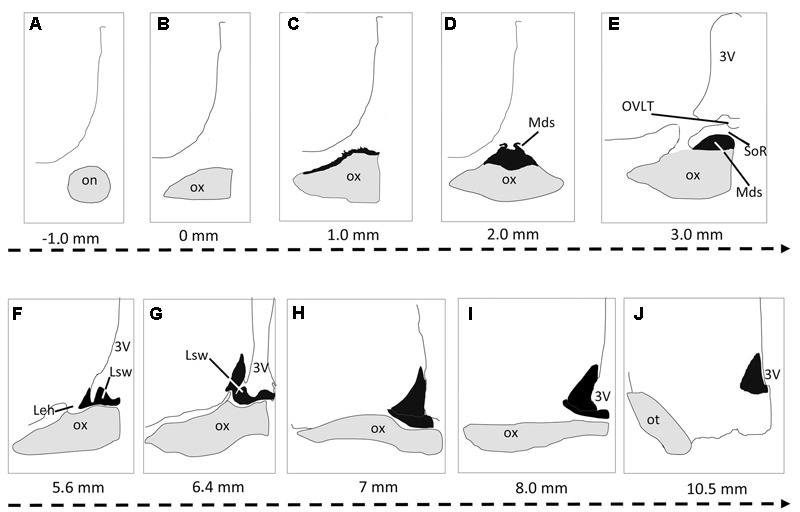
Schematic representation of the suprachiasmatic nucleus (SCN) organization in the dromedary. The serial drawings show the rostrocaudal extent of the SCN in coronal sections. The nucleus is represented in black; the optic nerve (on) optic chiasm (ox) and optic tract (ot) are in gray. The remaining areas are delineated by drawing lines representing the borders of the third ventricle, the preoptic area and the rest of the hypothalamus. The level of each section is indicated by the distance (mm) from the point of fusion of optic nerve (level 0: optic chiasm formation). Note that in the camel, the SCN is very long and divided into 3 parts: the rostral SCN (rSCN: **A–G**), the main SCN (mSCN: **H,I**) and retrochiasmatic or caudal SCN (cSCN: **J**). Note also that the rostral SCN is located outside the main mass of the hypothalamus lining the dorsal surface of the optic chiasm. 3V, third ventricle; Leh, lateral expansions of the hypothalamus; Lsw, lateral suprachiasmatic swellings of rSCN; Mds, median dome-shaped tissue of rSCN; OVLT, *Organum vasculosum* of the *lamina terminalis*; SoR, supraoptic recess.

**FIGURE 2:**
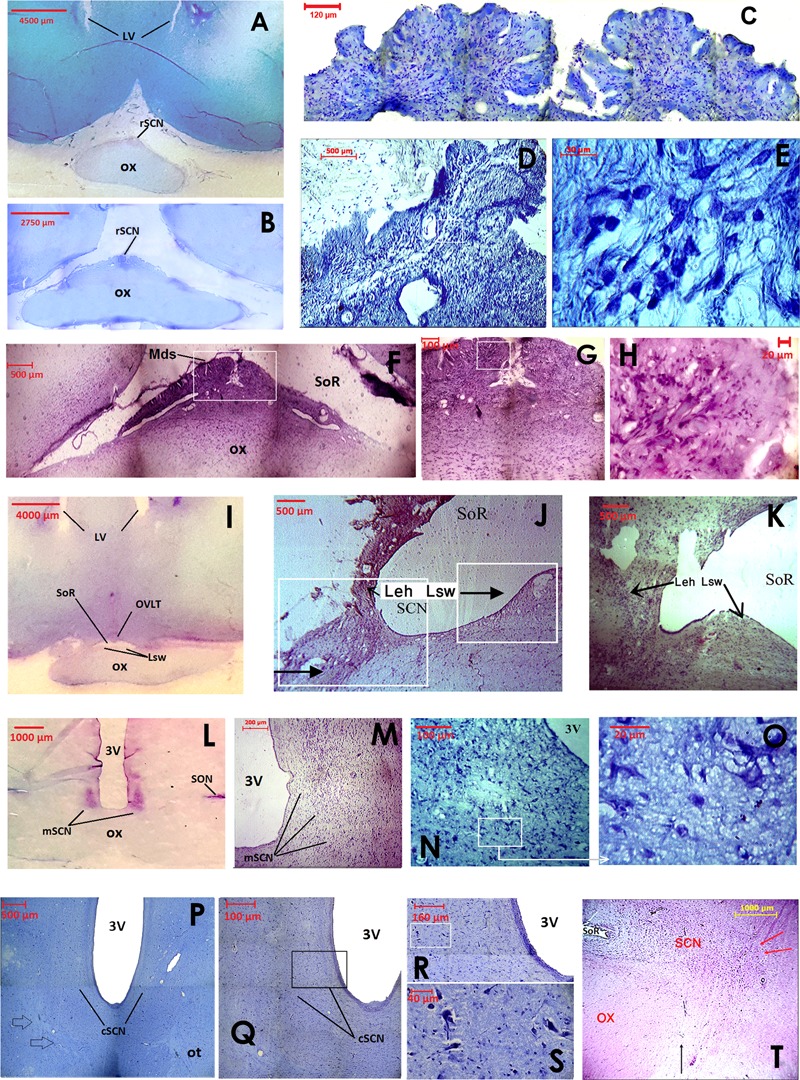
Images showing the cytoarchitecture and rostrocaudal organization of the camel SCN using classical histology staining of coronal sections. The rostral SCN (rSCN) is presented in **(A–H)**, the main SCN (mSCN) in **(I–O)**, the retrochiasmatic or caudal SCN (cSCN) in **(P–T)**. **(A)**: Low power view of toluidine blue-stained section at the level of preoptic area. The image shows the location of the rSCN. **(B)**: Montage of 15 images showing the location of the rSCN lying on the dorsal surface of the optic chiasm (ox). Toluidine blue staining. **(C)**: Montage of 12 images showing the peculiar morphology of the rSCN medially, juxtaposed to the dorsal surface of ox. Toluidine blue staining. **(D)**: Image in another representative animal showing the same shape of rSCN tissue as in **(C)**. Toluidine blue-stained section. **(E)**: Higher magnification of the field boxed in **(D)**, showing small-sized neurons of the rSCN stained with toluidine blue. **(F)**: Morphology of cresyl violet-stained rSCN at the middle level of its rostro-caudal extent. The image shows the median dome shaped tissue (Mds) of rSCN under the supraoptic recess (SoR). Montage of 6 images. **(G)**: Higher magnification of the field boxed in (F). Montage of 4 images. **(H)**: Cresyl-violet-stained neurons of rSCN. Higher magnification of the field boxed in **(G)**. **(I)**: Low power view of cresyl-violet- stained section of the anterior region of the hypothalamus. The image shows the location of the rSCN at its caudal level. Note that at these levels, the Mds tissue of rSCN is split into two lateral swellings of tissue (Lsw) under the SoR and the *Organum Vasculosum* of the *Lamina Terminalis* (OVLT). **(J,K)**: Images showing progressive changes of the Lsw from median to the lateral position. At their most lateral part, the Lsw fuse with the lateral expansions of the hypothalamus (Leh). This constitutes the beginning of the mSCN portion (Cresyl violet). **(L)**: Low power view of cresyl violet- stained section of the tuberal region of the hypothalamus. The image shows the location of the bilateral mSCN occupying the classical location within the hypothalamus at the ventral edges of the third ventricle (3V). **(M)**: Image of the mSCN from another representative camel (Cresyl violet). **(N)**: Image of the ventral part of the mSCN stained with toluidine blue. **(O)**: Higher magnification of the field boxed in (N) showing small-sized cells of the mSCN (Toluidine blue). **(P)**: Montage of 4 images (Toluidine blue) showing the location of cSCN in the caudal part of tuberal region of the hypothalamus. Note that at this level of coronal sections, the boundaries of the cSCN are difficult to delimit from surrounding regions. **(Q)**: Montage of 12 images (Toluidine blue) showing at higher magnification part of the image shown in **(P)**. **(R)**: Montage of 12 images (Toluidine blue) showing at higher magnification the field bordering the third ventricle in **(Q)**. **(S)**: Higher magnification of the field boxed in **(R)** (Toluidine blue) showing small-sized neurons of the cSCN. **(T)**: Sagittal section showing the rostrocaudal extent of the SCN in the camel (H&E). Note the rostral position of the rSCN under the SoR and also the retrochiasmatic extent of the cSCN (its limits are indicated by the red arrows) caudal to the ox (black arrow).

The SCN of the camel could be clearly distinguished from the surrounding hypothalamic nuclei and areas using Nissl staining. The nucleus appeared as a bilateral, confined aggregate of neurons, relatively long and extending rostro-caudally for 9.55 ± 0.10 mm (**Figure 1**). Its shape and location were complex and changed along the rostrocaudal axis. Based on histological and immunofluorescence findings, the SCN showed a distinct topography (**Figures 1, 2**), permitting us to subdivide the camel SCN into rostral or preoptic (rSCN), middle or main body (mSCN) and caudal or retrochiasmatic (cSCN) divisions.

Extending for 5.40 ± 0.12 mm, the rSCN is the largest division of SCN, and appears as a collection of neurons located outside the main mass of the hypothalamus, and was observed to protrude from the preoptic area (**Figures 1, 2A,B,F,I,J,K**). The most rostral part of this division is represented by a thin horizontal band of tissue laying on the dorsal surface of the optic chiasm, 1 mm from the point where the optic nerves fuse into their chiasm (**Figures 1C, 2A,B**). More caudally, the nucleus is located within and below the supraoptic recess (SoR) and ventral to the *organum vasculosum* of the *lamina terminalis* (OVLT). The rostral SCN division extends caudally and the supraoptic narrow tissue band becomes a dome-shaped tissue (Mds) at the midline (**Figures 1D,E, 2F,G**). Proceeding caudally, this Mds cell group divides into two lateral suprachiasmatic swellings (Lsw) jutting on either side into the SoR (**Figures 1F,G, 2I–K**). At this level, the OVLT is delineated ventrolaterally by two lateral expansions of the hypothalamus (Leh), which constitute a strand supporting the optic chiasm and contribute to form the SoR. Further caudally, the optic chiasm ascends dorsally and the supraoptic recess disappears (**Figures 1G,H**). The swellings of the rSCN become closer to the main mass of the hypothalamus, within which they gradually merge to fuse with the mSCN. Caudally and beyond these levels, the mSCN exhibits the characteristic triangular shape described in rodents (**Figures 1H,I, 2L–N**). Its total length is 2.09 ± 0.10 mm. In this location, the SCN is found bilaterally in the basal hypothalamus and is formed by dense neuronal aggregates, separated by the third ventricle. A thin layer of tissue, which persists within the boundaries of the optic chiasm, connects the two sides of the mSCN.

The cSCN is observed in the retrochiasmatic areas posterior to the optic chiasm (**Figure 2T**) and at the beginning of the optic tract. It extends rostrocaudally for 2.29 ± 0.13 mm. At this level, the nucleus is located dorsally to the arcuate nucleus (Arc). It is formed by periventricular neurons at the base of the third ventricle (**Figures 1, 2P–S**). Compared to the mSCN, the cell density of the cSCN diminishes at these levels. More caudally, the borders of this nucleus become progressively indistinct.

Measurements of cell soma diameters reveal that the cytoarchitecture of the camel SCN is heterogeneous. The majority of cell bodies are small-sized but there are also a number of large-sized neurons (**Figures 2F,N,O**). The small-sized neurons are predominant in the main body of the nucleus and also dorsally (**Figure 2H**), whereas the large-sized cell bodies are less numerous and located ventrally (**Figures 2R,S**). This distribution was largely confirmed by our immunofluorescence studies. The small-sized cells of the camel SCN present a mean diameter of 10.1 ± 0.1 μm (range: 5–13 μm). They are generally elongated, triangular or round-shaped. The large-sized cells in the ventral part of the SCN show a mean diameter of 26.5 ± 1.5 μm (with a majority of diameters ranging from 15 to 35 μm). Their somata display various irregular shapes. Immunofluorescence investigations show that the large-sized neurons are less numerous than the small-sized ones and are observed in the narrow supraoptic tissue band of the rSCN laying directly on the optic chiasm. Overall, the dromedary SCN displayed several populations of neurons, with different sizes, shapes and neurochemical phenotypes.

### VIP Immunoreactivity

#### Neuronal Cell Bodies

As expected, numerous neuronal cell bodies were VIP-ir in the camel SCN. The VIP-positive perikarya were confined within the SCN and were not found in the surrounding hypothalamus.

**FIGURE 3:**
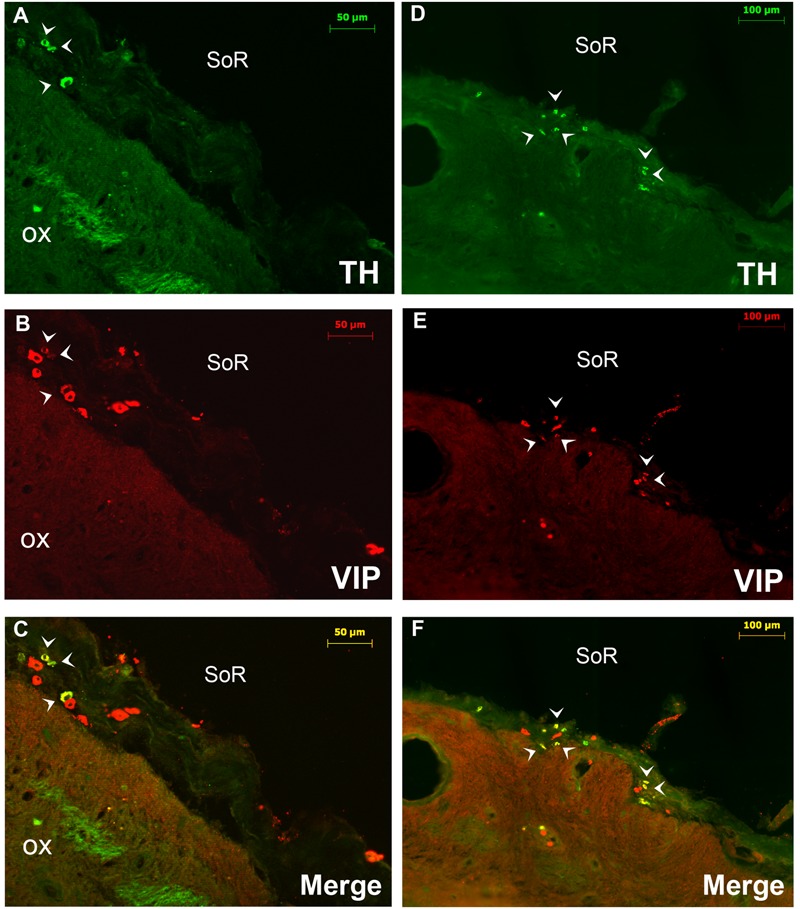
Double immunofluorescence of VIP (red) and TH (green) in the rostral SCN (rSCN) of the camel. **(A–F)** Represents respectively two coronal sections corresponding to the levels of panel (E) in **Figure 1**. Merge shows colocalization (arrowheads) of VIP and TH. ox, optic chiasm; SoR, supraoptic recess.

**FIGURE 4:**
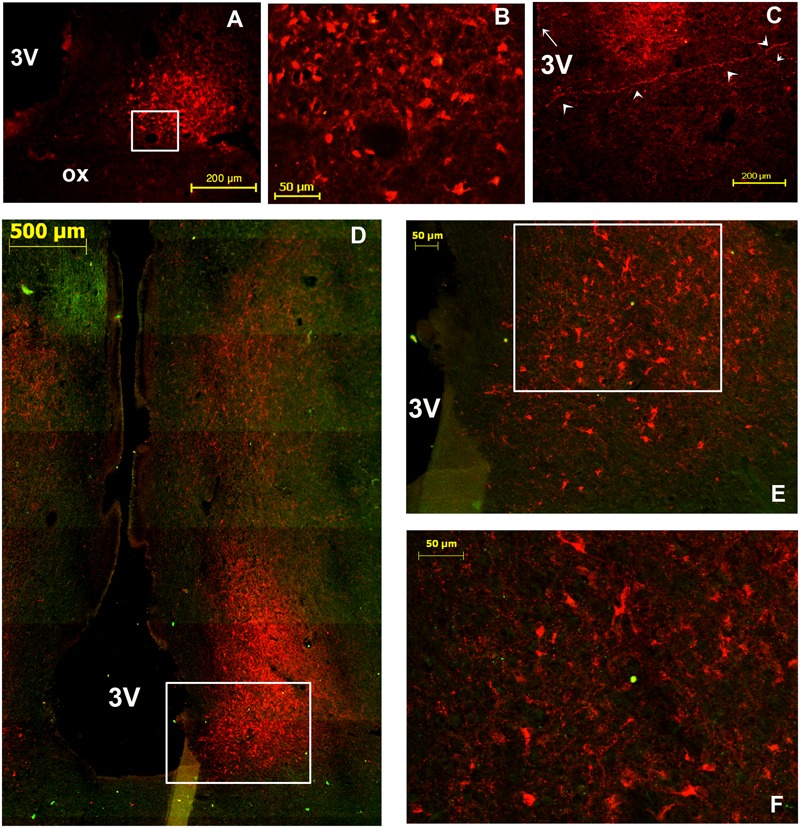
Immunofluorescent labeling of vasoactive intestinal polypeptide (VIP; red, **A–C**) and double immunolabelling **(D–F)** of VIP (red) and tyrosine hydroxylase (TH: green) in the main SCN (mSCN) of the camel. The coronal sections correspond to different levels of (H,I) of **Figure 1**. **(B)**: higher magnification of the field boxed in **(A)**. **(D)** Is a reconstruction of 18 images representing a merge of red (VIP) and green (TH) immunofluorescence in the camel hypothalamus. **(E)**: higher magnification of the field boxed in **(D)**. **(F)**: higher magnification of the field boxed in **(E)**, showing the VIP-ir neurons within the mSCN. Note that the VIP-ir cells form a cluster of small-sized neurons located ventrally in the rostral levels of mSCN **(A–C)**, whereas at the middle levels of the mSCN **(D–F)**, these neurons occupy almost the entire nucleus. VIP-ir fibers were branching up dorsally to dorsomedial (DMH) and paraventricular (PVN) nuclei and to other dorsal hypothalamic areas. ox, optic chiasm; 3V, third ventricle.

**FIGURE 5:**
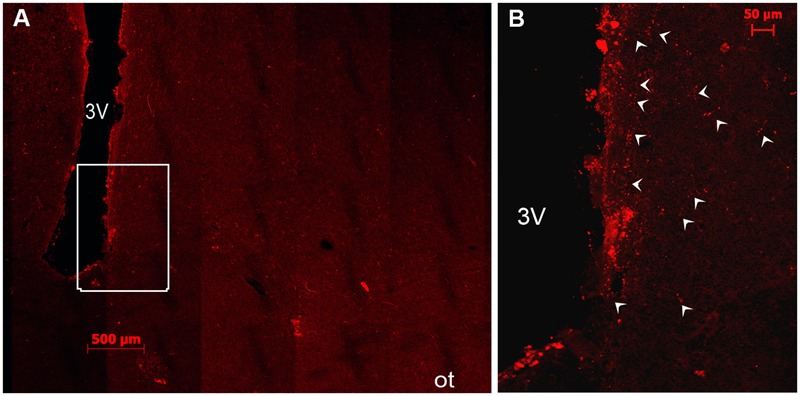
Immunofluorescent labeling of vasoactive intestinal polypeptide (VIP) in the caudal SCN (cSCN) of the camel. The coronal section corresponds to the level of panel (J) of **Figure 1**. **(A)**: montage of 25 images showing the VIP labeling in the hypothalamus of the camel; **(B)**: higher magnification of the field boxed in **(A)**. Note that at the cSCN levels there are very sparse VIP-ir neurons and fibers (arrows). 3V, third ventricle.

In the rSCN, the VIP-ir perikarya were clustered at the dorsal edge of the nucleus immediately below the SoR (**Figure 3**). These cells were round, with a mean diameter of 14.0 ± 0.8 μm (ranging 6–20 μm) and displaying a small soma with a narrow nucleus and a large cytoplasm (**Figures 3B,E**). These small-sized neurons occupied a dorsal position along the different levels of the rSCN. Thus, they were seen in the Mds and the Lsw SCN tissue. In these locations, the VIP-ir neurons gave rise to fibers directed toward the OVLT vessels. In the rostral levels of the mSCN (**Figures 4A–C**), the VIP-positive cells appeared first in the ventral region of the nucleus adjacent to the optic chiasm, and became concentrated to form a round, dense cluster of perikarya. More caudally, the VIP-ir-small-sized neurons were numerous and not restricted to the ventral surface (**Figures 4D–F**), and were widely distributed through a large area of the mSCN core body. The mSCN VIP-ir neurons were small-sized, with a mean diameter of 13.6 ± 0.5 μm (ranging from 9 to 18 μm), and displayed a small perikaryon, a relative narrow nucleus and a large cytoplasm. Their shape was predominantly multipolar. The VIP-ir neurons were sparsely distributed in the cSCN at this level (**Figure 5**).

Double immunostaining for VIP and the neuropeptides and enzymes we examined showed a colocalization only of VIP and TH in several neurons of the rSCN (**Figure 3**).

#### Fibers

The SCN of the camel showed numerous fine VIP-ir fibers with varicosities, generally uniform in size. These fibers were observed within the nucleus but also running out of the SCN toward the surrounding structures. They were observed at the rSCN level coursing laterally to the optic chiasm and dorsally to the OVLT vessels. In the mSCN, the VIP-ir fibers were first directed toward the dorsal part of the nucleus (**Figures 4A,D**). At the rostral levels of the mSCN, VIP-ir fibers were also directed toward the supraoptic nucleus (SON) (**Figure 4C**), while in the caudal part of the mSCN, these fibers constituted a dense network leaving the nucleus toward the dorsal hypothalamic area (**Figure 4D**). These VIP-ir fibers were observed branching dorsally to the paraventricular nucleus of the hypothalamus (PVN) and a large part of them changed their direction from the third ventricle to deviate laterally toward the hypothalamic dorsomedial nucleus, within which they formed an oblique dense plexus (**Figure 4D**). Furthermore, other thick-caliber periventricular fibers were observed to course dorsally, probably toward the subthalamic region. More caudally, in the cSCN, only some VIP-positive fibers were seen in the area of the nucleus and at the supraependymal level along the third ventricle wall (**Figure 5**).

### AVP Immunoreactivity

#### Neuronal Cell Bodies

**FIGURE 6:**
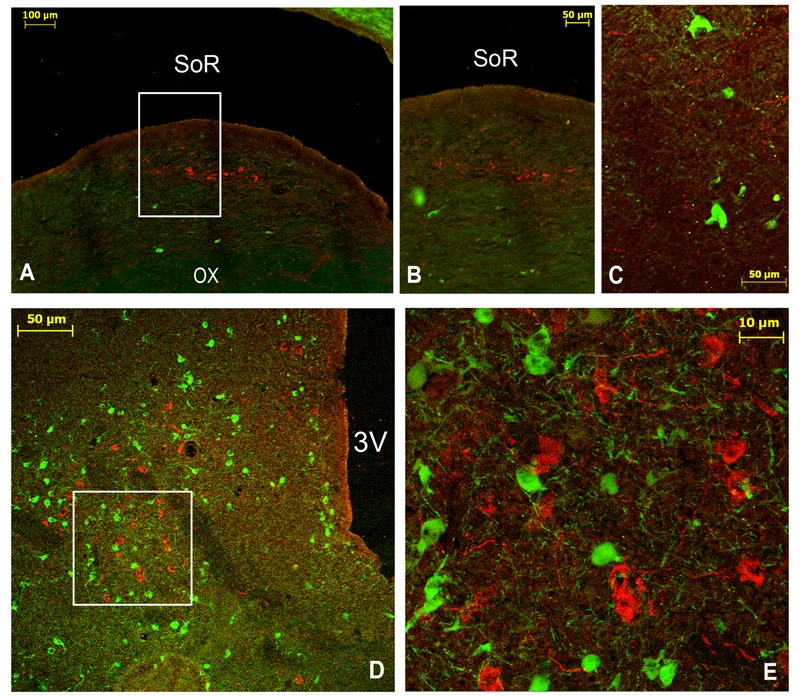
Double immunofluorescence of arginine-vasopressin (AVP) and tyrosine hydroxylase (TH) in the SCN of the camel. **(A–C):** Merge of AVP (green) and TH (red) in the rostral SCN (rSCN): levels of panel (E) of **Figure 1**. **(D,E):** Merge of double labeling of AVP (red) and TH (green) in the main SCN (mSCN): levels of (I) of **Figure 1**. **(A)** is a montage of 9 images; **(B)**: higher magnification of the field boxed in **(A)** showing AVP-ir perikarya in rSCN; **(C)**: image from the hypothalamus of another camel showing AVP neurons and TH fibers in the rSCN; **(D)**: distribution of AVP and TH neurons in the mSCN; **(E)**: higher magnification of the field boxed in **(D)**, showing AVP and TH neurons. Note the dorsal and lateral locations of AVP neurons in the mSCN and the lack of colocalization of AVP and TH in the same neurons. 3V, third ventricle; SoR, supraoptic recess.

AVP-ir neurons were observed at different levels of the rostrocaudal extent of the camel SCN. They were located at the ventral boundary of the rSCN (**Figures 6A,B**) and also at the dorsal edges and in the lateral parts of the mSCN (**Figure 6D**). In the remaining sections sampled from the mSCN, the AVP-ir perikarya were observed only in the dorsal portion of the nucleus above the ventral population of VIP-ir neurons. Caudally, only sparse AVP-ir neurons were observed.

The AVP-ir perikarya displayed an irregular shape, and a few of them were round-shaped (**Figures 6C,D**). They were the smallest neurons of the SCN, ranging from 5 to 15 μm in diameter with a mean diameter of 8.8 ± 0.4 μm. Their nuclei were large and they were mostly of the multipolar type (**Figure 6D**).

#### Fibers

Immunofluorescence revealed the presence of AVP-ir fibers in the camel SCN. Some of them were observed within the nucleus (**Figures 6C–E**), while the majority was seen to course from the SCN to surrounding hypothalamic areas. In addition to the SON, the densest network of AVP-ir fibers appeared to connect the SCN with the PVN. Other AVP-ir fibers were located along the periventricular edge of the third ventricle.

### OT Immunoreactivity

#### Neuronal Cell Bodies

**FIGURE 7:**
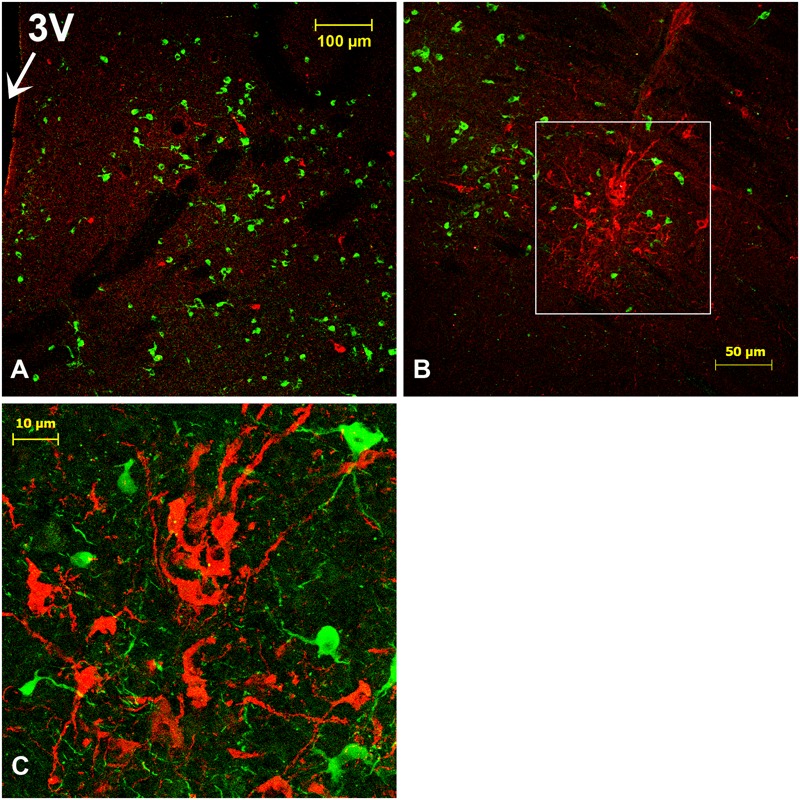
Oxytocin (OT)-immunoreactive (ir) neurons in the SCN of the camel. Merge of OT (red) and tyrosine hydroxylase (TH; green) immunofluorescence in the main SCN (mSCN): levels of panels (H) and (I) of **Figure 1**. **(A)**: OT-ir neuronal cell bodies scattered throughout the mSCN. **(B)**: image of the caudal level of the mSCN showing OT-ir neurons clustered in the ventrolateral part of the nucleus. **(C)**: Shows at higher magnification the field boxed in **(B)**, with OT-ir and TH-ir neurons in the mSCN. 3V, third ventricle.

OT-ir perikarya within the camel SCN were observed at different levels of the mSCN in coronal sections but neither in the rSCN nor in the cSCN (**Figure 7**). The OT-ir neuronal cell bodies were either scattered throughout the mSCN (**Figure 7A**) or clustered in the ventrolateral portion of the nucleus (**Figure 7B**). They were exclusively of small size with a diameter ranging from 5 to 16 μm and a mean diameter of 9.2 ± 0.5 μm. Their somal shape was irregular, sometimes with a round shape containing a small nucleus and a large cytoplasm. They were predominantly multipolar (**Figure 7C**).

Double immunofluorescence revealed that there was no co-localization of OT with either AVP or with the other neuropeptides studied. Overall, the density of OT neurons was lower than that of AVP-ir or VIP-ir neurons.

#### Fibers

OT-ir fibers were observed within the mSCN (**Figures 7A,B**), and also leaving the mSCN toward the surrounding area.

### TH Immunoreactivity

#### Neuronal Cell Bodies

**FIGURE 8:**
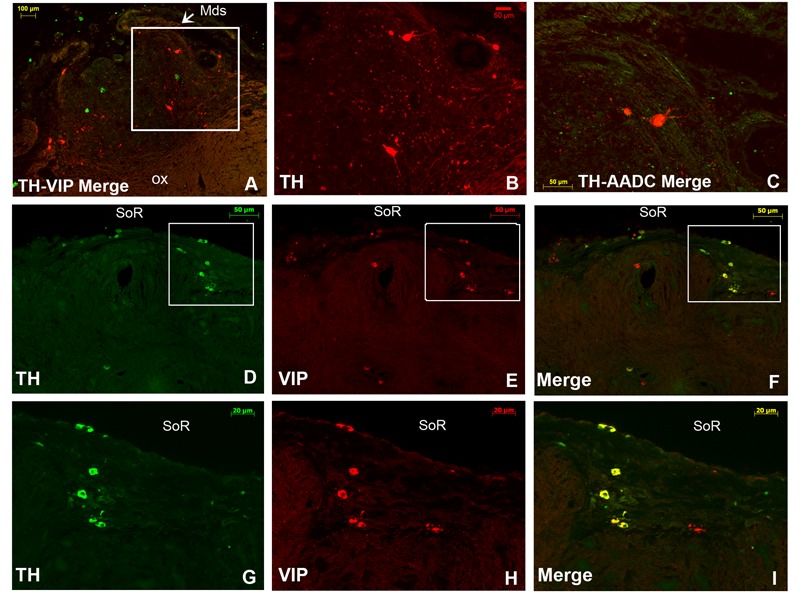
Double immunofluorescence of tyrosine hydroxylase (TH) and vasoactive intestinal polypeptide (VIP), and TH and aromatic L-amino acid decarboxylase (AADC) in the rostral SCN (rSCN) of the camel. **(A)**: montage of 12 images representing a merge of TH (red) and VIP (green) in the rSCN [levels of (D) of **Figure 1**]. **(B)**: higher magnification of the field boxed in **(A)** showing large-sized TH neurons in the ventral part of rSCN. **(C)**: merge of TH (green) and AADC (red) immunofluorescence, showing sparse AADC neurons in the rSCN of the camel which do not coexpress TH. **(D–F)**: double immunofluorescent labeling of TH (green) and VIP (red). The coronal sections correspond to levels of (E) in **Figure 1**. **(G,H,I)** Are higher magnifications of fields boxed in **(D,E,F)**, respectively. Note that at the dorsal levels of rSCN in **(D,G)**, the TH-ir neurons are small-sized and that the merge **(F,I)** shows that these neurons coexpress VIP. ox, optic chiasm; Mds, medial dome shaped tissue of rSCN; SoR, supraoptic recess.

**FIGURE 9:**
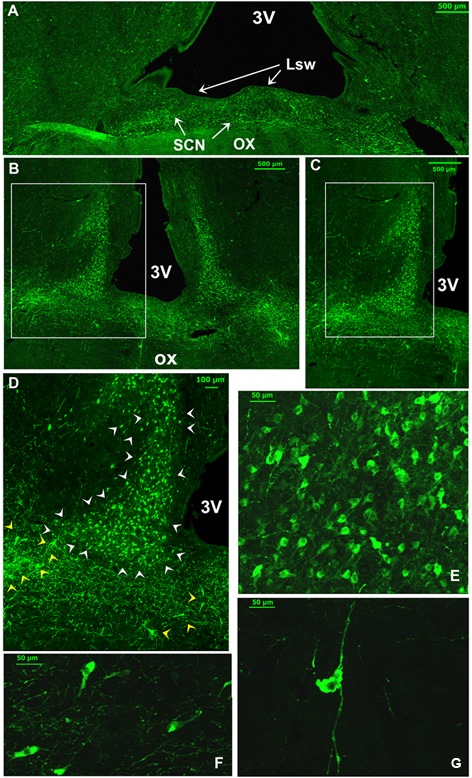
Illustration of two main populations of tyrosine-hydroxylase (TH)-immunoreactive (ir) neurons in the camel SCN. **(A)**: TH immunofluorescence in the most caudal levels of the rostral SCN (rSCN) and just prior the beginning of the main SCN (mSCN). Levels of coronal section correspond to panel (G) in **Figure 1**. Note at this level, as in the rostral part of rSCN, two TH-ir neuronal populations: dorsal small-sized and ventrolateral large neurons. **(B)**: TH immunofluorescence in the mSCN [levels of (H) of **Figure 1**). The montage (of 16 images) shows TH- immunolabelling in the mSCN of the two sides. **(C)** Shows at higher magnification, the field boxed in **(B)**, with TH-ir neurons in the mSCN. **(D)** Shows at higher magnification the field boxed in **(C)** with TH-ir neurons and fibers in the right mSCN. Note also at these levels the existence of two populations of TH-ir neurons: small-sized neurons (white arrowheads) which fill the mSCN except for the lateral and ventrolateral portions of the nucleus, and large-sized neurons (yellow arrowheads) in the ventral part of the mSCN with medial and lateral clusters of neurons. Large-sized neurons show intense immunofluorescence and are intermingled with a dense network of TH-ir fibers. **(E)**: dorsal TH-ir small-sized neuronal population. **(F,G)**: different types of the ventral large-sized TH-ir neurons.

**FIGURE 10:**
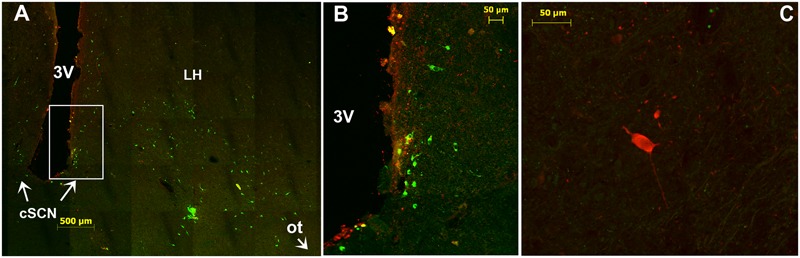
Immunofluorescence of tyrosine hydroxylase (TH) in the caudal SCN (cSCN). **(A)**: montage of 25 images showing merge of TH (green) and VIP (red) in the caudal part of the tuberal region of camel hypothalamus and cSCN. The coronal section corresponds to the levels of panel (J) in **Figure 1**. **(B)**: higher magnification of the field boxed in **(A)**. Note that in the cSCN the TH-ir perikarya are exclusively small-sized neurons, scattered and not very numerous. **(C)**: merge of TH (green) and AADC (red); showing rare, sparse AADC neurons in the cSCN of the camel and which do not coexpress TH. 3V, third ventricle; LH, lateral hypothalamus; ot, optic tract.

Several neuronal cell bodies were TH-immunopositive in the camel SCN. These neurons exhibited some peculiarities. First, at the level of the rSCN, TH ir-neurons were observed in different parts, especially in the Mds and Lsw tissue (**Figures 8A,B,E,H, 9A**). Indeed, in the Mds and especially in the Lsw (**Figure 8**), two populations of TH-ir neurons were revealed: a dorsal population which contained small neurons with a mean diameter of 16.1 ± 0.7 μm (ranging from 5 to 20 μm) juxtaposed to the SoR (**Figures 3, 8D–I, 9A**) and a ventral population which was instead formed by less numerous but more intensely immunopositive large-sized neurons with a mean diameter of 33.5 ± 6.1 μm (ranging from 25 to 39 μm) and lying on the dorsal surface of the optic chiasm (**Figures 8A,B, 9A**). The small-sized neurons were mostly unipolar and the ventral larger neurons were mostly bipolar or multipolar. These ventral large and dorsal small TH-ir neurons were also observed within the mSCN. We have observed in different sections through the mSCN both the ventral population of TH-ir large neurons, with a mean diameter of 39.8 ± 2.0 μm (ranging from 30 to 50 μm), and the dorsal population of small neurons with a mean diameter of 15.0 ± 1.0 μm (ranging from 5 to 22 μm) (**Figures 9B–G**). The small-sized TH-ir neurons were found in the most superficial part of the mSCN, whereas the ventral TH-ir large-sized neurons were located within the ventral extent of the mSCN in a medial and two lateral clusters of neurons (**Figures 9B–D**). The TH-ir ventral large neurons were of different shapes, mostly bipolar or multipolar but also of the unipolar and pseudo-unipolar types (**Figures 9D,F,G**), whereas most of the dorsal TH-ir small neurons were unipolar or bipolar (**Figure 9E**). More caudally, in the cSCN, the TH-ir perikarya were exclusively large-sized, with a mean diameter of 24.1 ± 1.5 μm (ranging from 11 to 28 μm), scattered and less numerous (**Figure 10**). The majority of these neurons were unipolar and located in a very medial and periventricular position.

Double immunofluorescence showed that TH-ir neurons did not express any immunoreactivities for the neuropeptides we examined (**Figures 4D–F, 6, 7**), except for VIP. In fact, only a few TH-ir dorsal small-sized neurons of the rSCN showed such colocalization (**Figures 3, 8D–I**). TH-ir neurons within the SCN did not express AADC (**Figures 8C, 10C**).

#### Fibers

The TH-ir fibers were observed at different levels of the SCN. In the rSCN, fibers were seen in Mds, the two Lsw, and in Leh-containing tissue (**Figures 8A–C, 9A**). Fibers were abundant ventrally at the junction of the optic chiasm. Caudally, some of these fibers appeared to course laterally to penetrate the SON. In the mSCN, the TH-ir fibers formed a dense network in the region of TH-ir large-sized neurons but also in the dorsal region containing small-sized neurons. These TH-ir fibers were very dense in the ventral part of the mSCN, forming a horizontal plexus delineating the optic chiasm (**Figures 9B–D**). At these levels, some fibers appeared to course laterally toward the SON. Other fibers had a vertical path with a ventral orientation and a ventrally penetrating trajectory in the optic chiasm. Furthermore, the ventral TH-ir large-sized neurons also extended fibers toward the dorsal population of TH-ir small-sized neurons and the lateral area of the nucleus devoid of TH-ir cell bodies (**Figures 9C,D**). Double immunofluorescence did not identify the chemical phenotype of neurons within this area targeted by TH-ir fibers. Caudally, in the cSCN, TH ir-fibers were less dense (**Figure 10B**).

### AADC Immunoreactivity

Immunolabelling of AADC was performed to identify whether the TH-ir neurons within the camel SCN are dopaminergic. The labeling demonstrated the presence of only rare, sparse AADC-ir neurons in the three parts of the SCN. Few labeled fibers were also observed. These neurons were large-sized and did not coexpress TH (**Figures 8C, 10C**).

### Met-Enk, 5-HT and NPY Immunoreactivities

**FIGURE 11:**
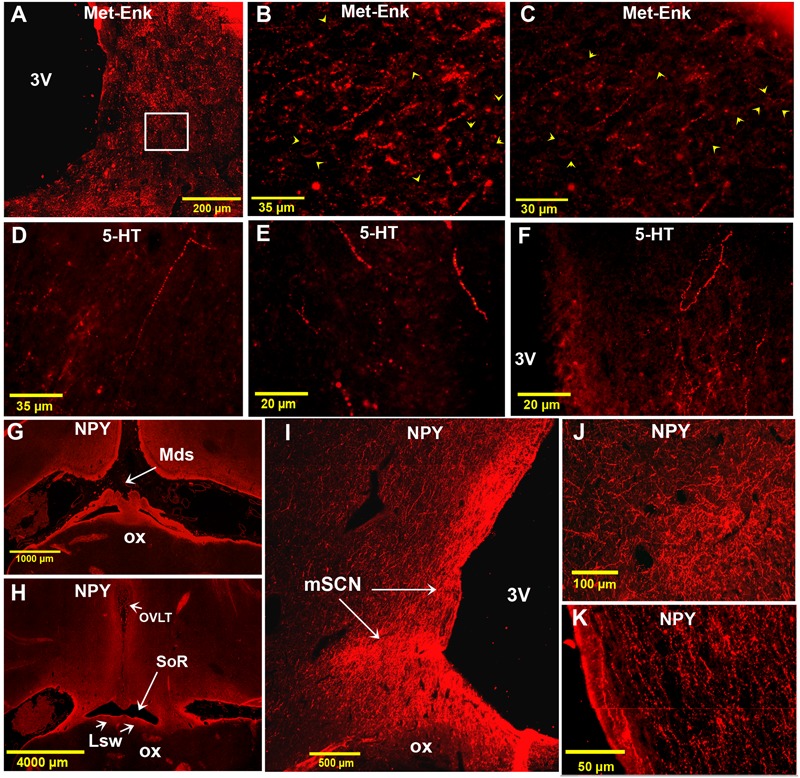
Immunofluorescence of met-enkephalin (Met-Enk), 5-HT and neuropeptide Y (NPY) in the camel SCN. **(A)**: montage of 70 images showing Met-Enk immunolabelling in a low power view of the main SCN (mSCN). **(B)**: higher power view of the field boxed in **(A)** showing Met-Enk-immunoreactive (ir) fibers and perikarya (arrowheads) in the mSCN. **(C)**: Image from another animal showing Met-Enk fibers and neurons (arrowheads) in the SCN. **(D)**: lateral 5-HT-ir long fibers descending ventromedially from the dorsal hypothalamic area toward the left mSCN. **(E)**: 5-HT-ir fibers descending ventrally from the dorsal hypothalamic area and lining the 3 V wall, toward the ventral right mSCN. **(F)**: Plexus of thick 5-HT-ir fibers in the mSCN. **(G)**: Montage of 16 images showing a low power view of the rSCN and the hypothalamus labelled for NPY. Level corresponds to (D) in **Figure 1**. **(H)**: Montage of 38 images corresponding to the level between panel (E) and (F) in **Figure 1** showing the rSCN and the hypothalamus labeled for NPY. **(I)**: Montage of 12 images corresponding to the level of panel (I) in **Figure 1** and showing NPY labeling in the mSCN. **(J)**: Montage of 4 images corresponding to level of panel (I) in **Figure 1**, showing NPY labeling in the mSCN. **(K)**: Montage of 2 images corresponding to level of (H) in **Figure 1**, note the dense NPY immunopositivity also at these levels.

A very dense network of Met-Enk-ir fibers was observed in the camel SCN. These fibers displayed numerous varicosities forming a plexus within the three divisions of the SCN and especially in the mSCN (**Figures 11A–C**). Furthermore, plexuses of Met-Enk-ir fibers were widely distributed at different levels in surrounding areas of the hypothalamus. Immunolabelling showed also that the camel SCN contains several Met-enk-ir neurons (**Figures 11B,C**).

Immunofluorescence did not reveal 5-HT-ir perikarya in the camel SCN. Instead, there were numerous 5-HT-ir fibers (**Figures 11D–F**), with a relatively thick caliber and non-uniform varicosities. Interestingly, these fibers were observed to descend laterally from the dorsal hypothalamic area and also lining the wall of the third ventricle toward the SCN (**Figures 11D,E**). These 5-HT-ir fibers were found to form a dense plexus within the mSCN (**Figure 11F**).

Examination of the different levels of the camel SCN showed that this nucleus did not contain NPY-ir neuronal cell bodies. However, a high density of NPY-ir fibers was found within the camel SCN (**Figures 11G–K**). These fibers were mainly varicose and formed a dense network in the mSCN, but were sparse in rSCN and cSCN. Compared to all the other neurochemically identified fibers observed in the present study, the most extensively distributed fibers in the SCN were those immunoreactive for NPY.

## Discussion

This study is the first undertaken on camel SCN neuroanatomy including its cartography, cytoarchitecture and chemoarchitecture. The results show that the SCN of the camel displays peculiarities regarding its size, extent, morphology and cytoarchitecture. Based on histological and immunohistochemical criteria, we subdivided the camel SCN into rostral, main and caudal divisions (rSCN, mSCN and cSCN; respectively). The location of the rSCN, organized as a collection of neurons protruding from the preoptic area outside the main mass of the hypothalamus, is unusual. The SCN at these levels is located below the SoR, lining the dorsal surface of that portion of optic chiasm that is still detached at these levels from the ventral borders of the brain. Despite the fact that such a conformation is uncommon in most studied species, a similar organization has been described in sheep (Tillet et al., 1989; Tessonneaud et al., 1994), cow (Lignereux, 1986), and in humans (Swaab et al., 1985; Hofman et al., 1988; Hofman and Swaab, 1989). Similarities with these species were also demonstrated with regard to the rostrocaudal morphological variations of the SCN. Indeed, structures comparable to the median dome-shaped tissue (Mds), lateral suprachiasmatic swellings tissue (Lsw), and lateral expansions of the hypothalamus (Leh); have been observed in the rSCN of the above-mentioned species. Caudally, the mSCN exhibits the usual triangular shape and location at the base of the third ventricle, dorsally to the optic chiasm, as classically described in rodents and several other mammals (Spiegel and Zweig, 1917; Bleier et al., 1979; van den Pol, 1980; Cassone et al., 1988; Vrang et al., 1995).

Due to the existence of its two rostro-caudal subdivisions, the rSCN and cSCN, which are lacking in most species, the dromedary SCN seems to be the largest studied thus far. This can be attributed to the fact that the SCN starts far rostrally outside the main mass of the hypothalamus and elongates caudally to the optic chiasm. The SCN extends in the camel for 9.55 mm, while in sheep and in humans (species which exhibit a similar SCN morphology), the total rostrocaudal length is much shorter (2.8–3.1 mm and 1.47 mm, respectively; Swaab et al., 1985; Swaab and Hofman, 1990; Tessonneaud et al., 1994). The rostrocaudal extent of the SCN in other mammals depends on the species but ranges between 0.60 and 2.15 mm (0.60 mm in the Syrian hamster, 0.9 mm in the rat, 0.76 mm in the guinea pig, 0.76 mm in the mouse, 0.80–1.0 mm in the squirrel monkey, 0.90 mm in the macaque monkey, 0.95–1.65 mm in five marsupial species, 1.74 mm in the domestic pig and 2.15 mm in the domestic cat) (van den Pol, 1980; Lydic et al., 1982; Cassone et al., 1988).

There are great differences in the general topography and cytoarchitecture of the SCN across species. It seems reasonable to argue that mammals can be divided in two groups concerning the morphology of the SCN: camel, sheep, cow and human (and maybe other species) with a relatively long and large SCN, protruding out of the hypothalamus in its rostral division, and other mammals (including rodents) with the SCN corresponding to more “classical” descriptions.

Based on retinal projections, size and shape criteria as well as neurochemical phenotypes, the SCN, especially in rodents, has been divided in two dorso-ventral subdivisions: the dorsomedial SCN or ‘shell’ and the ventrolateral SCN or ‘core’ (Abrahamson and Moore, 2001; Morin et al., 2006). In the present histological study, no such subdivisions were evident in the camel SCN. However, clear cytoarchitectural differences were identified with respect to the size of SCN neurons, with populations of dorsal parvicellular and ventral magnocellular neurons. This organization is largely confirmed by the immunofluorescence studies. In sheep, there is also no clear dorso-ventral subdivision of the SCN, but neurons are smaller in the ventral than in the dorsal region of the SCN (Tessonneaud et al., 1994). Neurons of the camel SCN are the smallest cells among those observed within the hypothalamus and adjacent areas. Nevertheless, the camel SCN contains neurons larger than those reported in sheep and other species, especially in the ventral magnocellular part, in which neurons are larger than 20 μm in diameter and can reach up to 50 μm. The largest somal diameters in the SCN of many species (including the domestic pig, domestic cat, mouse, guinea pig, rat, hamster and five marsupial species) do not exceed 12.5 ± 3.1 μm (measured in the ventrolateral division of the cat SCN), while in other species the diameter of SCN neurons ranges between 7.0 and 10.7 μm (van den Pol, 1980; Cassone et al., 1988).

The neurochemical organization of the camel SCN is different from that of the other species studied so far. In camel SCN, two populations of TH neurons were identified, ventral magnocellular or dorsal parvicellular. Their location, number, size and immunoreactivity intensity, suggests that they could have different roles. In the absence of a precise additional phenotyping of these neurons, it is difficult to further speculate on their role (s). To our knowledge, no similar findings were reported in the SCN of other mammals. Very few TH-ir neurons were observed to be homogeneously distributed in the SCN of sheep (Tillet et al., 1994). Moreover, in most rodents, this nucleus either lacks or contains only a few TH neurons. For example, in the rat, a few TH neurons were reported to be present only transiently in the developing brain (Battaglia et al., 1995; Ugrumov, 2013). In the Syrian hamster, only sparse TH-ir round-shaped cells were observed in the SCN, mostly outside the nucleus and lining its borders (Vincent, 1988; Novak and Nunez, 1998; Strother et al., 1998).

Tyrosine hydroxylase is the rate-limiting enzyme for the biosynthesis of catecholamines (dopamine, epinephrine and norepinephrine) and catalyzes the transformation of the amino acid L-tyrosine into L-DOPA (L-3, 4 Dihydroxyphenylalanine), which is then converted to dopamine by AADC (also called Dopa-decarboxylase). Within various animals, the AADC-ir neurons in the SCN seem to be more abundant than the TH neurons (Sheep: Tillet et al., 1994; rat: Jaeger et al., 1984; Inatomi, 1994; cat: Kitahama et al., 1988; house-shrew: Karasawa et al., 1992).

Regarding the puzzle of the relatively high density of TH-ir neurons we observed in the camel SCN, we have investigated their possible dopaminergic phenotype by testing colocalization with AADC. The double immunostaining showed that AADC-ir neurons are very rare and sparse within the nucleus, large-sized and never coexpress TH. This indicates that there are no catecholaminergic, especially no dopaminergic, neurons in the SCN of the camel. The lack of AADC expression within the TH-ir neurons indicates the synthesis of L-DOPA, but not dopamine, in these neurons. This raises several questions. L-DOPA itself has a role in mediating the release of neurotrophic factors that are important for the growth, survival and differentiation of neurons (Lopez et al., 2008; Malenka et al., 2009; Zigmond et al., 2012; Hiroshima et al., 2014). In the camel SCN, given the relatively high number of TH-neurons, L-DOPA could be involved in the survival and differentiation of neurons. A possible involvement of TH neurons in mediating circadian activity is also suggested by the colocalization of TH with VIP especially in rSCN neurons.

The presence of OT-ir neurons in the SCN has been reported briefly by El May et al. (1987) when studying the hypothalamo-hypophyseal axis of the camel. However, their given localization of the SCN and their description as a large extension from the optic chiasm to the pituitary stalk attachment were not very precise. In the present work, the camel SCN cartography is performed at high spatial resolution and results confirmed clearly that OT neurons are found within the mSCN and are either scattered throughout the nucleus or grouped ventro-laterally. The presence of OT-ir neurons within the SCN is unusual, and represents a peculiarity of the nucleus in the camel. Sofroniew and Weindl (1980) studied the neuropeptidergic content of the SCN in 13 species belonging to 6 mammalian orders (marsupials, rodents, lagomorphs, artiodactyls, carnivores and primates). In all of these animals, the SCN lacks OT-ir neurons, as confirmed in other studies (Sofroniew and Glasmann, 1981; Reuss et al., 1989; Caba et al., 1996). For the camel, a possible role of OT neurons in the modulation of the circadian clock activity cannot be excluded. This could be of particular interest in species such as the dromedary, that faces the problem of adaptation to the harsh environment of the desert and which has to adapt its physiology and circadian rhythms to this biotope.

The existence of vasopressinergic neurons in the camel SCN is consistent with reports in other mammalian species (Vandesande et al., 1975; Vandesande and Dierickx, 1975; Sofroniew and Weindl, 1980; Card et al., 1981; Tillet et al., 1989; Kalsbeek and Buijs, 1992; Kikusui et al., 1997; Abrahamson and Moore, 2001; Kalsbeek and Buijs, 2002). However, the distribution of these neurons is different in the camel SCN compared to the animals studied thus far. They occupy different locations on the rostrocaudal extension of the nucleus and appear more caudally in a dorsomedial position forming a population equivalent to the “shell” described in rodents (Ibata et al., 1999; Moore et al., 2002; Morin et al., 2006; Nascimento et al., 2010). The distribution of AVP-ir neurons in the camel SCN, as well as their relatively high number, seem to be a peculiarity of a species living in arid and desert biotopes. In the jerboa (*Jaculus orientalis*), a semi-desert rodent living in the same latitudes as the dromedary, AVP neurons in the SCN are intensely immunostained, and are located in the dorsomedial and ventromedial but also dorsal, dorsolateral and ventral portions of the SCN (Lakhdar-Ghazal et al., 1995a). Across all species studied to date, the AVP neurons located in the dorsomedial SCN play a crucial role in the development and distribution of circadian signals (for a review, see Reghunandanan and Reghunandanan, 2006) and this could also be the case for the camel SCN. Furthermore, studies have shown that the amount of vasopressin release in the SCN (Kalsbeek et al., 1995) and its mRNA levels (rat: Larsen et al., 1994, mice: Smith and Carter, 1996, Siberian hamster: Duncan et al., 1995) show daily variations with a diurnal acrophase. This rhythmicity is maintained under constant conditions (Yamase et al., 1991; Cagampang et al., 1994), demonstrating its circadian origin.

A relatively large number of VIP-ir neurons were found in the camel SCN. In the present investigation, the highest density of these neurons within the camel hypothalamus was observed in the SCN. Similar observations have been made in other mammalian species (Card et al., 1981; Stopa et al., 1984; Cassone et al., 1988; Tessonneaud et al., 1994; Abrahamson and Moore, 2001; Moore et al., 2002). It is well known that the ventrolateral subdivision of the SCN receives direct retinal afferents (Moore, 1973; Cassone et al., 1988; Ibata et al., 1989; Abrahamson and Moore, 2001), contacting VIP neurons (Ibata et al., 1989). Moreover, VIP expression (mRNA and peptide) in the SCN exhibits a nycthemeral rhythm depending on the LD cycle (Albers et al., 1990; Ibata et al., 1993; Shinohara et al., 1993; Yang et al., 1993; Larsen et al., 1994). In the camel SCN, the most rostral VIP-ir neuronal population, forming a cluster of cells in the ventral part of the mSCN, is similar in its shape and location to the findings reported in other species. Although no data on retinal projections is available in the camel, due to its location this group of VIP neurons could serve as the target of such projections also in the camel.

In rodents, VIP neurons project to the dorsal AVP neurons in the SCN (Jacomy et al., 1999) to regulate the activity of the clock by light (Harmar et al., 2002). Our findings suggest that VIP neurons in the camel SCN also project to the AVP neurons located dorsally. We also observed a dense plexus of VIP-ir fibers directed to the hypothalamic area above the SCN, which seem to reach the PVN and the dorsomedial nucleus, as reported in several other species (Kalsbeek et al., 1993; Saper et al., 2005). The neurophysiological significance of such dorsal projections could be related to the well-known modulatory role of VIP on AVP neurons (Watanabe et al., 1998; Jacomy et al., 1999; Maywood et al., 2006) and thus on clock activity. AVP neurons, in turn, would distribute the modulated circadian message to other structures in the brain.

In the jerboa, the SCN content of VIP shows seasonal variations (Lakhdar-Ghazal et al., 1992) which seems to be related to the photoperiod and the effect of sex hormones (Oukouchoud et al., 2003). Such mechanisms remain to be investigated in the camel. However, in view of data on the variation in the duration of melatonin secretion in this species (El Allali et al., 2005, 2008) and the existence of seasonal breeding activity, it seems reasonable to suppose that the photoperiod could modulate VIP expression in the camel SCN.

The present results show that the NPY-ir fibers were the densest in the camel SCN among the other neurochemically identified fibers investigated in this study. The NPY fibers form a dense plexus in different parts of the camel SCN, and especially in the mSCN. Such a high density of NPY fibers coming from the IGL has been previously described in the rodent SCN (Moore et al., 1984; Ueda et al., 1986; Sabatino et al., 1987; Card and Moore, 1989; Morin et al., 1992; Lakhdar-Ghazal et al., 1995b; Jacob et al., 1999; Menet et al., 2001; Abrahamson and Moore, 2001) and these fibers contact VIP neurons (Ibata et al., 1988; François-Bellan and Bosler, 1992). This innervation is reportedly involved in non-photic synchronization mechanisms (Challet et al., 1996, 1997; Juhl et al., 2007). The neuroanatomical pathways and the entrainment by the IGL are thus well demonstrated in rodents, but remain to be fully understood in the camel, sheep and most non-human primates. In non-human primates, a complex of NPY neurons, the pregeniculate nucleus, is equivalent to the IGL of rodents, but does not send efferents to the SCN (Moore, 1989; Chevassus-au-Louis and Cooper, 1998). Likewise, the SCN of the sheep harbors only sparse NPY-ir fibers (Tillet et al., 1989). The present findings show a dense plexus of NPY fibers in the camel SCN, but a combination of immunohistochemistry and tract tracing is necessary to address its origin from the IGL.

The camel has to adapt its physiology and to anticipate changes in its harsh environment by integrating the most important environmental cues, mainly the environmental temperature and LD cycles. The density of NPY-ir fibers in the camel SCN may reflect the integration of non-photic signals, correlated with an important non-photic entrainment of the circadian clock represented by the daily cycle of environmental temperature (El Allali et al., 2013).

The present findings also demonstrate a dense plexus of Met-Enk-ir fibers in the camel SCN. Met-Enk innervation of the SCN has also been reported in others species, including: mouse (Abrahamson and Moore, 2001), sheep (Tillet et al., 1989; Tessonneaud et al., 1994) and Syrian hamster (Morin et al., 1992). In this latter species, Met-Enk fibers originate from neurons located in the IGL (Morin and Blanchard, 1995) and participate in photic transmission and clock synchronization (Harrington and Rusak, 1986; Pickard et al., 1987; Edelstein and Amir, 1999; Juhl et al., 2007).

Our data for the camel SCN demonstrate that 5-HT immunopositive fibers cross the hypothalamus, especially the periventricular areas, toward the SCN. A high density of 5-HT fibers in the SCN has been reported for several species (Ueda et al., 1983; Abrahamson and Moore, 2001). These fibers constitute a third major set of afferents to the SCN and are involved in the transmission of non-photic stimuli to this nucleus. Serotonergic modulation of the SCN is well characterized in the Syrian hamster (Azmitia and Segal, 1978; Meyer-Bernstein and Morin, 1996; Hay-Schmidt et al., 2003; Vrang et al., 2003). Moreover, in some species (rat and cat), direct retinal projections to the raphe nuclei were identified. Photic information could therefore also reach the SCN indirectly through 5-HT fibers. The existence of a significant 5-HT innervation in the camel SCN could be related to the particular adaptation of this species to its biotope thus requiring pathways combining non-photic and photic entrainment.

In most mammals, the relative importance of photic versus non-photic entrainment of the circadian clock is not fully understood. In addition to LD entrainment, several non-photic factors can synchronize the circadian clock. These stimuli may be behavioral, dietary or other environmental factors such as environmental temperature, for which little information is available to date. It appears that the unusual morphology of the camel SCN (both in terms of its length and shape) and the uncommon existence of TH and OT neurons, in addition to the presence of a dense innervation of NPY, Met-Enk and 5-HT fibers, reflect collectively the importance of this nucleus in the circadian adaptation of the camel to its harsh biotope. The synchronization of the circadian clock in this species both by photic and non-photic cues deserves special study. Under experimental conditions (El Allali et al., 2013), we demonstrated that both entrainments occur in the camel: synchronization by the LD cycle and by the daily cycle of environmental temperature. Neuroanatomical interactions in the camel brain between different pathways for photic and non-photic entrainment are also very likely and warrant further investigation.

## Ethics Statement

There were no animal sacrifices to carry out this study. The work was conducted by using camels’s brains. Samples were taken as animals were slaughtered to provide meat for public consumption. The study was in conformation with the Hassan II Agronomy and Veterinary Institute of Rabat and Moroccan Ministry of Agriculture recommendations which are in accordance with international ethical standards (Touitou et al., 2006).

## Author Contributions

KEA, AC, PP, and NL-G conceived and designed the work; KEA, MA, and MO performed brains sampling; KEA performed immunohistochemical labeling experiments; KEA and MP performed image acquisition and quantification of immunofluorescence; KEA, EC, and PP prepared the manuscript; MA, MP, MO, EC, ME, NL-G, AC, and PP revised and approved the final review.

## Conflict of Interest Statement

The authors declare that the research was conducted in the absence of any commercial or financial relationships that could be construed as a potential conflict of interest.
